# Two-Dimensional Layered Nanomaterial-Based One-Dimensional Photonic Crystal Refractive Index Sensor

**DOI:** 10.3390/s18030857

**Published:** 2018-03-14

**Authors:** Jitendra B. Maurya, Alexandre François, Yogendra K. Prajapati

**Affiliations:** 1 Department of Electronics and Communication Engineering, Motilal Nehru National Institute of Technology Allahabad, Allahabad 211004, Uttar Pradesh, India; jitendra.maurya06@gmail.com; 2 School of Engineering and Future Industry Institute (FII), University of South Australia, Mawson Lakes, Adelaide SA 5095, Australia; Alexandre.Francois@unisa.edu.au; 3 Institute for Photonics and Advanced Sensing (IPAS) and School of Physical Sciences, University of Adelaide, Adelaide SA 5005, Australia

**Keywords:** one-dimensional, photonic crystal, refractive index, sensitivity, sensor

## Abstract

One-dimensional photonic crystal (1DPC) sensors have emerged as contenders for traditional surface plasmon resonance sensors, owing to their potential for the detection of bigger molecules and particles due to their higher interaction volume in the sensing medium. Two-dimensional layered nanomaterials, most notably graphene and dichalcogenides (e.g., MoS_2_, MoSe_2_, WS_2_, and WSe_2_), have shown higher refractive index sensitivity because of their absorption as well as adsorption property. The proposed configuration of 1DPC presented consists of alternate layers of the aforementioned nanomaterials and silicon. The performance parameters, namely the sensitivity, resolution, quality factor, and the evanescent field penetration depth, are calculated and compared with 1DPC having poly methyl methacrylate (PMMA) in place of silicon. Increased shift in resonance angle and quality factor are observed by replacing PMMA with silicon, but at the cost of decreased resolution. Further, our results show that although the sensitivity and quality factor of the 1DPC sensor is less than that of the conventional surface plasmon resonance sensor (SPR) with a gold thin film, it has much higher resolution and penetration depth to make it suitable for large molecules.

## 1. Introduction

**Figure 1:**
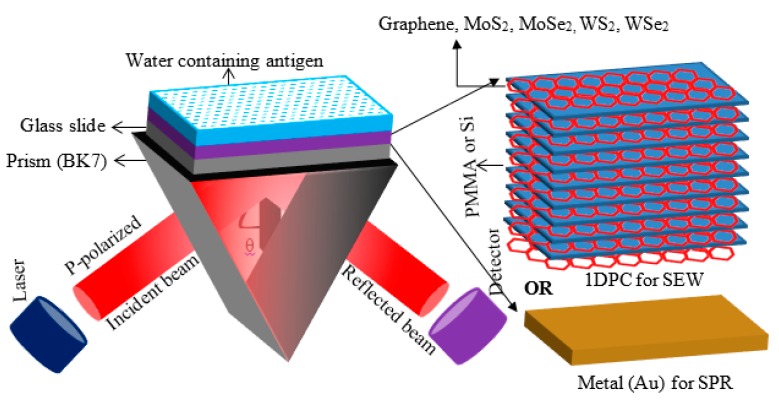
Schematic of the prism-based structure of the Au-surface plasmon resonance (SPR) sensor and the proposed one-dimensional photonic crystal (1DPC) sensor.

The quantification and characterization of biomolecular interactions is of critical importance for a wide range of applications ranging from healthcare to more fundamental research in proteomics and genomics. While there are several techniques fitted for that particular purpose, label-free sensing methods, especially those relying on measuring changes of the refractive index (RI), such as surface plasmon resonance (SPR) [1], Braggs gratings [2], and whispering gallery modes [3] to name a few, have gained prominence in the research community. This is not only due to their sensing performances but also their ability to conduct real-time measurements, providing critical information on binding constants which are otherwise not possible to obtain. SPR is by far the simplest of these methods, requiring in most cases a simple reflectivity measurement of a p-polarized monochromatic light source onto a plasmonic material (i.e., gold, silver, copper, aluminum), typically through a prism to match the propagation constant of the incident light source with the propagating plasmons, as shown in the Figure 1. However, SPR sensors suffer from the damping of light intensity caused by the metal absorption, which eventually degrades the sensor’s performance due to the broadening of the resonance curve [4]. Moreover, a limited number of materials can fulfil the criteria for generating a surface plasmon wave (SPW), which can be tuned at limited optical wavelengths [4]. Furthermore, the SPW evanescent field at the interface of the metal layer and sensing medium varies the temperature and composition of the sensing medium, which can subsequently vary the sensing medium RI [5]. Also, the shorter penetration depth (PD) of the SPW evanescent field, less than 300 nm, makes the SPR unsuited for large molecules such as mitochondria (~500 nm) [6]. In contrast to SPW, surface electromagnetic wave (SEW)- or Bloch wave-based one-dimensional photonic crystal (1DPC) sensors have much narrower resonance curves due to low loss in photonic crystals, which improves the resolution [7]. SEWs are non-radiative electromagnetic modes which can be excited from IR to near-UV ranges depending on the selection of dielectric materials and suitable design of 1DPCs [2]. Furthermore, 1DPCs exhibit lower optical loss compared to plasmonic materials, thus enhancing the coupling between SEW and incoming light. Moreover, lower loss in 1DPCs may provide a higher intensity SEW, leading to much sharper resonance dips and high surface electromagnetic fields responsible for the higher PD in the adjacent sensing medium.

1DPCs are made of photonic bandgap (PBG) materials in which the dielectric constants vary with a regular periodicity only in the perpendicular direction of the stacking of layers, prohibiting light propagation. 1DPCs show metal-like behavior inside the PBG [8]. Hence, 1DPCs have an effective dielectric constant whose real part is negative, which supports the SEW at frequencies within the PBG [9]. Hence, the principle of operation of 1DPC sensors is similar to SPR sensors but with PBG material in place of the metal film. Since the extinction coefficients of dielectric materials are much lower than those of metals, the reflectance curves of 1DPCs are much narrower, resulting in enhanced sensing performances [7]. Moreover, by the appropriate selection of the RI and thickness of the layers, 1DPCs can be tuned to almost any wavelength. In addition, by choosing the thermo-optic coefficients compensating materials carefully, the temperature variation at the interface can be minimized to a great extent [10].

Two-dimensional (2D) nanomaterials are attracting attention for their applications in biosensing because of their large surface to volume ratio and direct bandgap compared to the indirect bandgap of bulk materials [11]. The high surface to volume ratio of 2D layered transition metal dichalcogenides (2D-LTMDC) offers a larger contact area to attach the analyte. The photons absorption in direct bandgap materials are higher than that in indirect bandgap materials, requiring extra phonons to energize the electrons to exceed the intermediate state in the indirect bandgap material [12]. Moreover, nanomaterials like as graphene and dichalcogenides (i.e., MoS_2_) are capable of adsorbing biomolecules such as ssDNA thanks to π-stacking bonding [13] or through van der Waals forces [14], respectively.

The absorbance of 2D-LTMDC is wavelength-dependent and, at 633 nm, the order of absorbance for the considered nanomaterials is as follows: graphene < WS_2_ < WSe_2_ < MoSe_2_ < MoS_2_ [15]. The materials with lower absorbance values are suitable for sensing applications. Hence, the sensitivity order of these layered nanomaterials should be: graphene > WS_2_ > WSe_2_ > MoSe_2_ > MoS_2_. However, in the case of 1DPCs this order of sensitivity may be disturbed because reflection [16] or transmission [17] depends on the periodicity and thickness of the composite layers.

In this article, the general analytical analysis of the performance parameters characterizing the 1DPC refractive index sensors, i.e., sensitivity, resolution, quality factor, and the evanescent field penetration depth, are evaluated. The sensing performances of 1DPCs having either silicon or poly methyl methacrylate (PMMA) and different considered layered nanomaterials; graphene, WSe_2_, WS_2_, MoSe_2_, or MoS_2_, are then discussed and compared with a conventional gold-coated supporting SPR.

## 2. Proposed 1DPC Structure

The schematic of the prism-based gold SPR (Au-SPR) sensor and 1DPC sensor are shown in Figure 1. The operating wavelength (λ) of 633 nm is considered throughout this manuscript. At this wavelength, the refractive indices of the BK7 prism, glass plate, graphene, MoS_2_, MoSe_2_, WS_2_, WSe_2_, silicon, PMMA, and water are 1.515, 1.515 [7], 3 + 1.1491*i* [11], 5.0947 + 1.2327*i* [15], 4.6226 + 1.0063*i* [15], 4.8937 + 0.3124*i* [15], 4.5502 + 0.4332*i* [15], 3.916 [15], 1.49 [7], and 1.33, respectively, and thicknesses are semi-infinite, semi-infinite, 0.34 nm, 0.65 nm, 0.70 nm, 0.80 nm, 0.70 nm, 470 nm, 470 nm, and semi-infinite, respectively. For the Au-SPR sensor, a 45-nm thin gold layer having an RI of 0.14330 + 3.6080*i* is deposited onto the glass slide [11].

For 1DPC-based sensors architecture, the gold thin film is replaced by nine bi-layers of nanomaterials and Si, plus an additional layer of the considered nanomaterial on the top of the 1DPC behaving as an affinity layer for subsequent biomolecules immobilization. The refractive index of the sensing medium is varied from 1.33 to 1.40 to evaluate the performance characteristics, mimicking the adsorption of biomolecules onto the sensor’s surface.

## 3. Results and Discussion

The mathematical expression for reflection intensity can be obtained by solving Fresnel’s equations for the generalized N-layer model using the transfer matrix method (TMM) [7]. In the angular interrogation, the minimum reflection intensity (Rmin) is obtained at the maximum excitation of SEW, and the incidence angle corresponding to this Rmin is known as the resonance angle (θ_res_). Rmin will occur at the incidence angle greater than the critical angle (θ_c_) between the prism and water, i.e., $\theta \mathrm{res}>\theta_{c}=\sin^{-1}\left(n_{c}/n_{\mathrm{prism}}\right)=\;61.39{}^{\circ}$ [7]. The beam width (BW) of the reflectance curve is calculated as shown by Maurya et al. [18]. The resolution (detection accuracy) (1/BW) [19] and quality factor (S/BW) [20], where S is the sensitivity (S = ∆θ_res_/∆n), of the measurement can be enhanced by decreasing the BW of the reflectance curve. For a good sensor, all of these performance parameters should be as high as possible.

**Figure 2:**
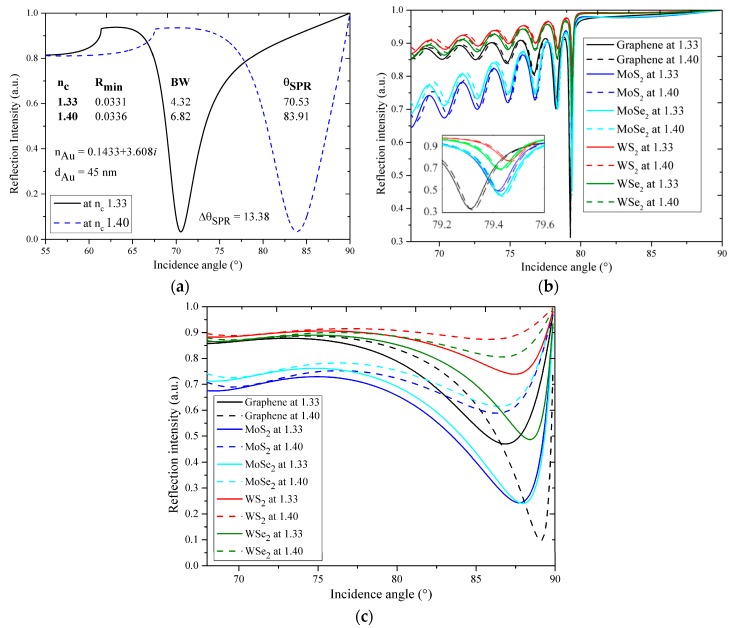
Reflectance curves calculated with surrounding refractive index of 1.33 and 1.40 for (**a**) Au-SPR sensor; (**b**) 1DPC with PMMA; and (**c**) 1DPC with silicon.

In Figure 2a‒c, the reflectance curves of the Au-SPR sensor and for different cases of both of the constituent materials of 1DPCs are plotted for a surrounding refractive index of 1.33 and 1.44. It is observed from Figure 2a‒c that all of the reflectance curves have resonance angles greater than the critical angle, i.e., 61.39°. It can also be seen from Figure 2a that for the Au-SPR sensor, the θ_res_, Rmin, and BW at 1.33 are 70.53, 0.03, 4.31, and those at 1.40 are 83.91, 0.03, 6.82, and the corresponding ∆θ_res_ is 13.38, which are arranged in Table 1.

**Figure 3:**
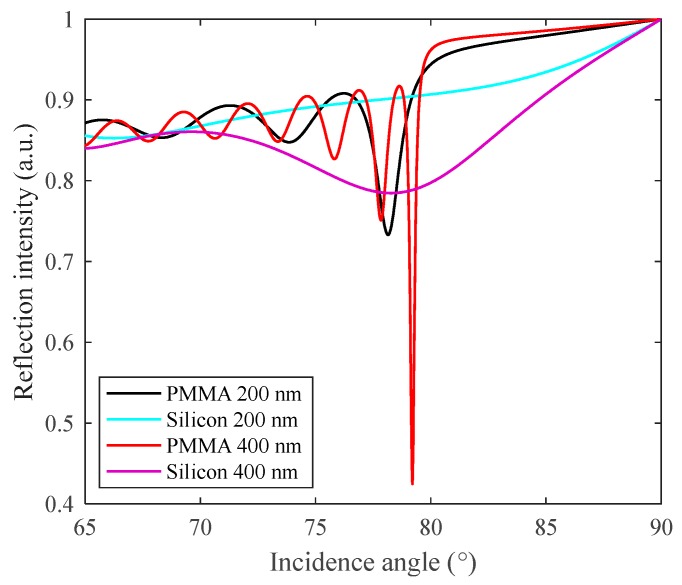
Reflectance curves of 1DPC having the first constituent material as graphene and different thicknesses of the second constituent material, either PMMA or silicon.

The Figure 2b,c show the calculated reflectance curves for 1DPCs with different materials considered, i.e., graphene, MoS_2_, MoSe_2_, WS_2_, WSe_2_, and PMMA or silicon as the intermediate layer, respectively. The performance defining parameters, i.e., ∆θ_res_, Rmin, and BW, of these reflectance curves are dependent on the thickness and RI of the nanomaterials, which are arranged in Table 1. The calculated values of S, resolution, and quality factor are also arranged in this table for a better comparison between different 1DPC and Au-SPR sensor architectures. Several dips (resonances) in Figure 2b may appear as per the combination of the refractive index and thickness of any constituent material, as previously observed by Sreekanth et al. [7], also shown in Figure 3. Out of these resonances, the deepest dip beyond the critical angle is considered as the main resonance.

From Figure 2b,c it can be observed that the BW for all of the 1DPC samples considered is predominantly influenced by the nature of the intermediate layer (PMMA or silicon), while the nanomaterials involved have very little to no influence, except for graphene. The BW of the PMMA-based 1DPCs is typically very low, compared to the silicon-based 1DPCs, which is desired for achieving both high resolution and quality factor. However, this also affects the refractive index sensitivity in the opposite direction, with the sensitivity being significantly reduced for the PMMA-based 1DPCs compared to the silicon-based ones. From Table 1, it can be seen that PMMA-based 1DPCs are almost insensitive to the change of the sensing medium RI. Replacing PMMA with silicon results in a significant refractive index sensitivity (S = ∆θ_res_/∆n) increase, as shown in Figure 2c, which compares the best-performing reflectance curves of 1DPC architecture comprising graphene with either PMMA or silicon as the intermediate layer. This is likely due to the high RI of silicon, which enhances the SEW [20]. However, at the same time BW is also increased, degrading both the resolution and quality factor.

Out of the different nanomaterials investigated, as mentioned, graphene exhibited by far the best sensing performances. While this is not necessarily obvious when using PMMA as an intermediate layer, which tends to average the sensing performances between the different nanomaterials investigated, it becomes obvious with the silicon-based 1DPCs. The quality factor, a figure of merit representing the RI detection limit, is about 10.66 RIU^−1^ for graphene/silicon 1DPC, almost twice that predicted for other nanomaterials. However, comparing this value with what is achievable with Au-SPR does not favor even the best-performing 1DPC, for which the quality factor is about one third that of Au-SPR.

**Figure 4:**
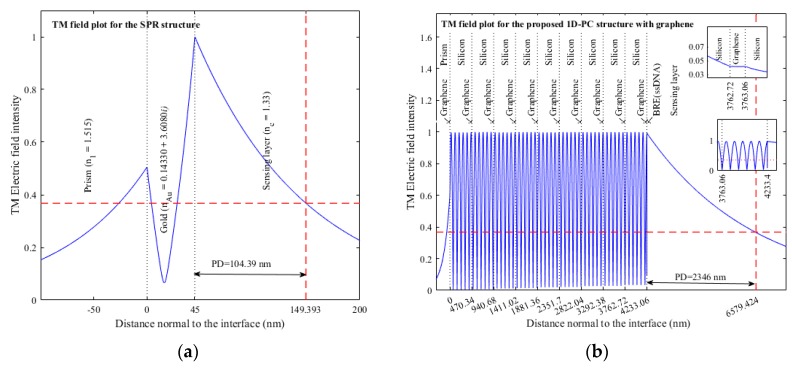
Electric field distribution along the distance normal to the stacking of layers for p-polarized light for (**a**) Simple SPR; (**b**) 1DPC [7].

The electric field distribution along the distance normal to the stacking of layers is plotted in Figure 4a,b for p-polarized light for the Au-SPR and graphene/silicon 1DPC sensors, respectively. The PD is defined as the distance travelled by the field normal to the layer in the sensing medium at which the field intensity decays to 1/e (37%). The calculated PD for the Au-SPR and graphene/silicon 1DPC sensors are 104.39 nm and 2346 nm, respectively, signifying that the interaction volume of field in the sensing medium is larger in the case of the graphene/silicon 1DPC sensor. Hence, although the quality factor for the SPR sensor is three times higher than that for the graphene/silicon 1DPC sensor, as seen from Table 1, the 1DPC sensor has a much higher PD which could be more suitable for sensing large biomolecules of a size from 50 nm to 2346 nm as well as cells e.g., lysosomes (200~500 nm), mitochondria (~500 nm), and secretory vesicles (50~200 nm) [21].

It was mentioned in the introduction of this article that materials having lower absorbance levels are suitable for sensing applications. Hence, the sensitivity order should be: graphene > WS_2_ > WSe_2_ > MoSe_2_ > MoS_2_, which is the opposite of the absorbance order; graphene < WS_2_ < WSe_2_ < MoSe_2_ < MoS_2_ [15]. However, in the case of 1DPC this order of sensitivity may be disturbed because of the reflection [16] or transmission [17] spectrum of the sensor, which depends on the pitch and thickness of the stacked layers. The obtained sensitivity for the case of silicon is: graphene > WSe_2_ > WS_2_ > MoSe_2_ > MoS_2_. Here, the disturbed order only affects the placement of WS_2_ and WSe_2_. Although the absorption of WSe_2_ > WS_2_, the sensitivity of 1DPC in the case of WS_2_ and WSe_2_ follows the same order as that for the absorption, i.e., WSe_2_ > WS_2_. This may be because of the lower thickness of WSe_2_ (0.70 nm) compared WS_2_ (0.80 nm).

We also identified the combination of graphene and silicon as the best-performing 1DPC, although its sensing performances are still lower than standard gold SPR. However, the graphene/silicon 1DPC benefits from a much higher evanescent field penetration, making such a platform highly advantageous for detecting large macromolecules/particles. Furthermore, this work did not take into account graphene’s structural nature, which exhibits a very high surface to volume ratio, potentially enabling immobilization by a much larger number of molecular probes, such as antibodies and DNA for subsequent target immobilization, which might be highly beneficial for reaching higher sensing performances.

## Figures and Tables

**Table 1:** Arrangement of obtained value of the minimum refractive intensity (Rmin) and beam width (BW) at 1.33 and 1.40, and ∆θ_res_ corresponding to ∆n = 1.40 − 1.33 = 0.07. On the basis of these values the calculated values of S, resolution, and quality factor are also arranged.

Constituent Material		Rmin (a.u.)		BW (Deg.)		∆θ_res_ (°)	S (°/RIU)	Resolution (/°)		Quality Factor (S/BW) (RIU^−1^)	
First Layer	Second Layer	1.33	1.40	1.33	1.40			1.33	1.40	1.33	1.40
Graphene	PMMA	0.32	0.31	0.13	0.14	0.008	0.11	7.69	7.14	0.85	0.79
MoS_2_		0.49	0.48	0.06	0.09	0.009	0.13	16.67	11.11	2.17	1.44
MoSe_2_		0.44	0.43	0.08	0.09	0.009	0.13	12.50	11.11	1.62	1.44
WS_2_		0.76	0.76	0.08	0.09	0.008	0.11	12.50	11.11	1.38	1.22
WSe_2_		0.68	0.68	0.09	0.09	0.008	0.11	11.11	11.11	1.22	1.22
Graphene	Silicon	0.49	0.06	6.19	3.31	2.47	35.29	0.16	0.30	5.70	10.66
MoS_2_		0.21	0.58	5.51	4.91	1.45	20.71	0.18	0.2	3.76	4.22
MoSe_2_		0.21	0.61	5.25	5.0	1.53	21.86	0.19	0.2	4.16	4.37
WS_2_		0.72	0.87	5.17	4.09	1.70	24.29	0.19	0.24	4.70	5.94
WSe_2_		0.44	0.80	4.32	4.94	1.85	26.43	0.23	0.20	6.12	5.35
SPR-Au		0.03	0.03	4.31	6.82	13.38	191.14	0.23	0.15	44.35	28.03

## References

[1] Homola J., Yee S.S., Gauglitz G. (1999). Surface plasmon resonance sensors: Review. Sens. Actuators B Chem..

[2] Sreekanth K.V., Zeng S., Shang J., Yong K.T., Yu T. (2012). Excitation of surface electromagnetic waves in a graphene-based Bragg grating. Sci. Rep..

[3] Reynolds T., Riesen N., Meldrum A., Fan X., Hall J.M.M., Monro T.M., François A. (2017). Fluorescent and lasing whispering gallery mode microresonators for sensing applications. Laser Photonics Rev..

[4] Sinibaldi A., Danz N., Descrovi E., Munzert P., Schulz U., Sonntag F., Dominici L., Michelotti F. (2012). Direct comparison of the performance of Bloch surface wave and surface plasmon polariton sensors. Sens. Actuators B Chem..

[5] Konopsky V.N., Alieva E.V. (2007). Photonic crystal surface waves for optical biosensors. Anal. Chem..

[6] Jian A. (2013). Resonant Optical Tunneling Effect for Refractive Index Sensor Applications;. Ph.D. Thesis.

[7] Sreekanth K.V., Zeng S., Yong K.T., Yu T. (2013). Sensitivity enhanced biosensor using graphene-based one-dimensional photonic crystal. Sens. Actuators B Chem..

[8] Joannopoulos J.D., Meade R.D., Winn J.N. (1995). Photonic Crystals: Molding the Flow of Light.

[9] Shinn M., Robertson W.M. (2005). Surface plasmon-like sensor based on surface electromagnetic waves in a photonic band-gap material. Sens. Actuators B Chem..

[10] Michelotti F., Descrovi E. (2011). Temperature stability of Bloch surface wave biosensors. Appl. Phys. Lett..

[11] Yang G., Zhu C., Du D., Zhu J., Lin Y. (2015). Graphene-like two-dimensional layered nanomaterials: Applications in biosensors and nanomedicine. Nanoscale.

[12] Dinh J.Q., He S., Qu J., Coquet P., Yong K.T. (2016). Sensitivity Enhancement of Transition Metal Dichalcogenides/Silicon Nanostructure-based Surface Plasmon Resonance Biosensor. Sci. Rep..

[13] Tang Z., Wu H., Cort J.R., Buchko G.W., Zhang Y., Shao Y., Aksay I.A., Liu J., Lin Y. (2010). Constraint of DNA on functionalized graphene improves its biostability and specificity. Small.

[14] Zhu C., Zeng Z., Li H., Li F., Fan C., Zhang H. (2013). Single-layer MoS2-based nanoprobes for homogeneous detection of biomolecules. J. Am. Chem. Soc..

[15] Dong N., Li Y., Feng Y., Zhang S., Zhang X., Chang C., Fan J., Zhang L., Wang J. (2015). Optical limiting and theoretical modelling of layered transition metal dichalcogenide nanosheets. Sci. Rep..

[16] Aly A.H., Ismaeel M., Abdel-Rahman E. (2012). Comparative study of the one dimensional dielectric and metallic photonic crystals. Opt. Phot. J..

[17] Wu K., Gu W., Wu C., Ma J., Ma X. (2014). Study on the Optical Transmission Properties of One-dimensional Photonic Crystal of MoS_2_. Adv. Mater. Res..

[18] Maurya J.B., Prajapati Y.K. (2017). A Novel Method to Calculate Beam Width of SPR Reflectance Curve: A Comparative Analysis. IEEE Sens. Lett..

[19] Srivastava T., Jha R., Das R. (2011). High-performance bimetallic SPR sensor based on periodic-multilayer-waveguides. IEEE Photonics Technol. Lett..

[20] Maurya J.B., Prajapati Y.K., Singh V., Saini J.P., Tripathi R. (2015). Performance of graphene–MoS_2_ based surface plasmon resonance sensor using Silicon layer. Opt. Quantum Electron..

[21] Ventra M., Evoy S., Heflin J.R. (2006). Introduction to Nanoscale Science and Technology.

